# Ongoing Radiographic Response for Over 20 Months in Metastatic Cancer Without Continued Immune Checkpoint Inhibitor (ICI) Dosing

**DOI:** 10.7759/cureus.80126

**Published:** 2025-03-06

**Authors:** Liyan Mazahreh, Farah Mazahreh, Mazin Safar

**Affiliations:** 1 Internal Medicine, University of Arkansas for Medical Sciences, Little Rock, USA; 2 Hematology and Medical Oncology, University of Arkansas for Medical Sciences, Little Rock, USA

**Keywords:** duration of treatment, immune-checkpoint inhibitors, immuno-chemotherapy, immunological response, metastatic non-small cell lung cancer

## Abstract

Non-small cell lung cancer (NSCLC) frequently presents with overt metastatic spread, portending a negative prognosis. Conventional treatments include chemotherapeutic agents and molecularly targeted and/or immunotherapeutic agents. Most responders to systemic chemotherapy in metastatic disease experience progression shortly after treatment discontinuation and it is very unusual for a patient to continue to manifest ongoing regression of malignant lesions unless treatment is continued. Moreover, responses occur early during therapy, typically within two to four months, and rarely continue beyond that time frame.

In this study, we describe a 67-year-old man with stage IV spindle cell cancer (initially diagnosed as NSCLC) who demonstrated ongoing radiographic regression over 20 months after receiving only a single dose of an immune checkpoint inhibitor (ICI) without any additional systemic therapy. Conventional approaches would have continued the ICI and credited any ongoing response to multiple doses. However, this case emphasizes that short exposure to ICI may be sufficient in select circumstances. Given that the five-year survival rate for stage IV non-small cell lung cancer (NSCLC) has historically been below 5%, observing such a durable response highlights the potential for more individualized immunotherapy strategies.

## Introduction

Non-small cell lung cancer (NSCLC) frequently presents with overt metastatic spread, which generally confers a dismal prognosis [1]. In metastatic disease, most responders to chemotherapy relapse soon after therapy ends [2]. Responses are also traditionally expected to appear early in treatment - within the first few months - and seldom continue to deepen if therapy is discontinued.

In the era of immune checkpoint inhibitors (ICIs), clinicians commonly maintain treatment for prolonged periods, assuming that ongoing exposure is needed for sustained immune-mediated tumor control [3]. This case challenges that assumption. A 67-year-old man with stage IV spindle cell lung cancer experienced a 20-month progression-free interval after just a single dose of ICI, with no other anticancer treatment. Histopathological analysis ultimately confirmed a metastatic spindle cell component, diverging from a typical primary adenocarcinoma of the lung.

Conventional approaches would have persisted with ICI dosing, thereby crediting long-term remission to continued therapy. In contrast, this single-dose scenario underscores the possibility that short ICI exposure can prompt a robust and durable immune response [4]. These observations may inform future strategies that reduce treatment burden, toxicity, and costs, given that they are validated by broader studies.

## Case presentation

A 67-year-old male originally presented in 2016 with stage IIIA NSCLC (adenocarcinoma) treated by right middle lobectomy, followed by adjuvant carboplatin/paclitaxel and postoperative radiotherapy (PORT). In 2022, he developed metastatic disease in the right lung, liver, and brain. An upper and lower GI endoscopy was performed due to microcytosis, which suggested a possible gastrointestinal primary, but no lesions were identified.

**Figure 1:**
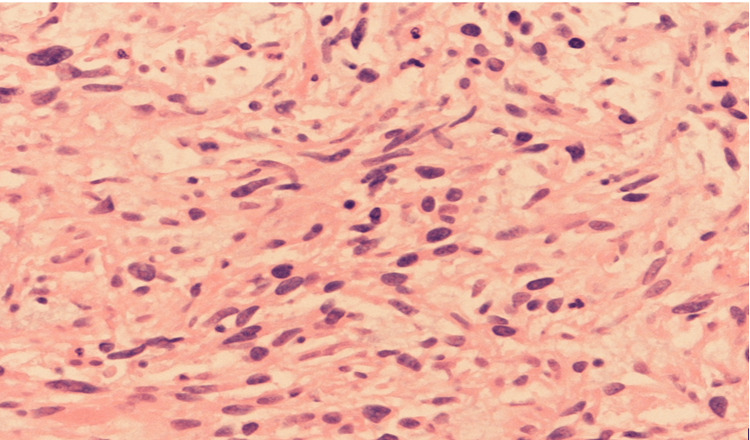
Histopathology of liver biopsy showing metastatic spindle cell carcinoma on H&E stain. The liver biopsy was reviewed in conjunction with the divisions of pulmonary and soft tissue pathology. Sections demonstrated a spindle cell neoplasm with significant mitotic activity and focal necrosis. Immunohistochemistry showed the tumor cells positive for AE1/AE3 and CK8/18, but negative for Alk-1, CK7, CK20, CDX-2, and CD117. CD163 highlighted scattered macrophages, and SMA was non-specific. These features support metastatic spindle cell carcinoma compatible with a pulmonary primary rather than a classic adenocarcinoma phenotype. PD-L1 testing on the spindle cell lesion was inconclusive due to limited tissue. No definitive biomarkers could be correlated with this prolonged ICI response. ICI: immune checkpoint inhibitor

After stereotactic radiation to the single brain metastasis, the patient received infusional 5-fluorouracil/oxaliplatin (FOLFOX) empirically. Histopathological analysis of liver lesions identified a spindle cell carcinoma likely pulmonary in origin despite atypical features (Figure 1). Because of a high proliferative Ki-67 index (90%), the patient received nine weekly doses of docetaxel. However, the disease displayed only modest shrinkage (approximately 10-15%).

**Figure 2:**
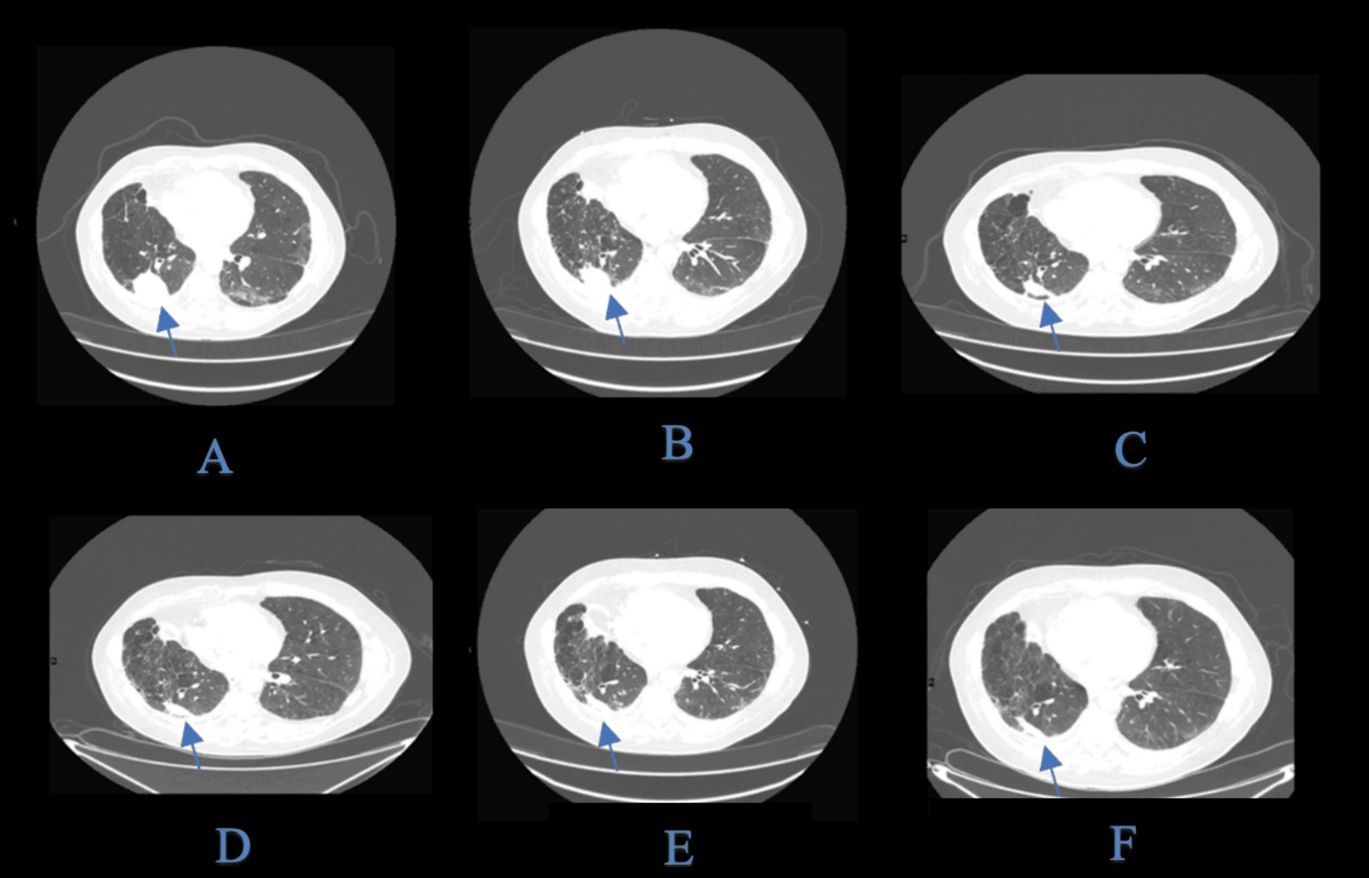
Sequential CT images of the lung index lesion. Similar pattern of regression was noted in the liver metastasis without the development of new lesions (arrows). An initial CT scan in April 2022 revealed marked metastatic disease in the right lung and liver prior to treatment. After chemotherapy and a single dose of pembrolizumab in August 2022, early tumor regression was observed. By November 2022, further shrinkage of the metastatic lesions was noted, with no new sites identified. In March 2023, there was continued regression of pulmonary and hepatic disease, reflecting a deepening response. By January 2024, radiographic imaging showed minimal disease. As of November 2024, over 20 months post-ICI, the disease remained stable, with no progression or new metastases, and no additional therapy required. ICI: immune checkpoint inhibitor

In August 2022, a single dose of pembrolizumab (200 mg IV) was administered (Figures 2A-1F). Radiographic imaging soon demonstrated more pronounced regression in both lung and liver lesions than had been achieved by chemotherapy alone. The response deepened over the subsequent months without any further anticancer therapy. As of his most recent CT scan in November 2024 - over 20 months after the single ICI dose - he continues to exhibit stable disease, with no new metastatic lesions. He has not reported any immune-related adverse events.

## Discussion

The patient’s remarkable 20-month response without continued ICI therapy raises questions about the duration of immunotherapy needed for persistent benefit [5]. Traditional protocols in metastatic NSCLC often maintain ICI until unacceptable toxicity or progression, partly inherited from chemotherapeutic “treat to progression” strategies [6]. This study underlines that tumor regression can continue beyond early imaging windows and even in the absence of further treatments.

Possible mechanisms of ongoing response

Immunologic Memory and T-cell Activation

Once activated, T-cells, including memory subsets, can mount prolonged surveillance against residual tumor cells. This is consistent with data showing that effector T-cell expansion peaks early after ICI initiation, potentially obviating further doses [3,7].

Docetaxel Priming

Cytotoxic chemotherapy reduces the proliferative fraction of malignant cells, potentially enhancing the effector-to-target ratio [8]. Docetaxel may also induce immunogenic cell death and diminish immunosuppressive populations, thus creating a more favorable tumor microenvironment.

Neutralizing Antibodies vs. Lasting Efficacy

Emerging studies show that neutralizing anti-pembrolizumab antibodies does not always negate efficacy, suggesting that early T-cell reprogramming may be sufficient in some patients to sustain a durable response [5].

Tumor Biology Variability

Spindle cell carcinoma may present unique immunological features. Although it remains unclear whether tumor genetics or microenvironment specifics contribute to this case, it appears that minimal ICI exposure was enough to drive a profound, enduring remission [9].

Clinical implications

Our case challenges the necessity of prolonged, uninterrupted ICI therapy. While it is an isolated observation, it exemplifies the potential for short-course immunotherapy to reduce costs, toxicity, and patient burden if validated in larger cohorts. It also accentuates the role that chemotherapy can play in “priming” tumors for immunotherapy success, though more rigorous mechanistic and biomarker-driven research is needed to identify ideal candidates for abbreviated ICI [10].

## Conclusions

This remarkable case has shown continuous regression of metastatic lesions for over 20 months in a patient with advanced NSCLC after discontinuation of therapy. This observation calls for further studies to delineate the biological underpinnings of extended remission in ICI-treated patients. Such investigations could yield new strategies for individuals who cannot tolerate prolonged immunotherapy or who may not require it. However, we underscore that this report is primarily hypothesis-generating. Validation via randomized trials - including those exploring high proliferative tumors, the role of combination chemotherapy, and biomarker-guided patient selection - remains essential before any changes to the standard of care can be recommended.
